# Impact of frequent cerebrospinal fluid sampling on Aβ levels: systematic approach to elucidate influencing factors

**DOI:** 10.1186/s13195-016-0184-z

**Published:** 2016-05-19

**Authors:** Bianca Van Broeck, Maarten Timmers, Steven Ramael, Jennifer Bogert, Leslie M. Shaw, Marc Mercken, John Slemmon, Luc Van Nueten, Sebastiaan Engelborghs, Johannes Rolf Streffer

**Affiliations:** Janssen Research & Development, a division of Janssen Pharmaceutica N.V., Beerse, Belgium; SGS Life Science Services, Antwerp, Belgium; Janssen Research & Development, LLC, Raritan, NJ USA; University of Pennsylvania Medical Center, Philadelphia, PA USA; Reference Center for Biological Markers of Dementia (BIODEM), Institute Born-Bunge, University of Antwerp, Antwerp, Belgium; Department of Neurology and Memory Clinic, Hospital Network Antwerp (ZNA) Middelheim and Hoge Beuken, Antwerp, Belgium

**Keywords:** Aβ peptides, Alzheimer’s disease, Catheterization, Cerebrospinal fluid, Sampling frequency

## Abstract

**Background:**

Cerebrospinal fluid (CSF) amyloid-beta (Aβ) peptides are predictive biomarkers for Alzheimer’s disease and are proposed as pharmacodynamic markers for amyloid-lowering therapies. However, frequent sampling results in fluctuating CSF Aβ levels that have a tendency to increase compared with baseline. The impact of sampling frequency, volume, catheterization procedure, and ibuprofen pretreatment on CSF Aβ levels using continuous sampling over 36 h was assessed.

**Methods:**

In this open-label biomarker study, healthy participants (*n* = 18; either sex, age 55 − 85 years) were randomized into one of three cohorts (*n* = 6/cohort; high-frequency sampling). In all cohorts except cohort 2 (sampling started 6 h post catheterization), sampling through lumbar catheterization started immediately post catheterization. Cohort 3 received ibuprofen (800 mg) before catheterization. Following interim data review, an additional cohort 4 (*n* = 6) with an optimized sampling scheme (low-frequency and lower volume) was included. CSF Aβ_1–37_, Aβ_1–38_, Aβ_1–40_, and Aβ_1–42_ levels were analyzed.

**Results:**

Increases and fluctuations in mean CSF Aβ levels occurred in cohorts 1–3 at times of high-frequency sampling. Some outliers were observed (cohorts 2 and 3) with an extreme pronunciation of this effect. Cohort 4 demonstrated minimal fluctuation of CSF Aβ both on a group and an individual level. Intersubject variability in CSF Aβ profiles over time was observed in all cohorts.

**Conclusions:**

CSF Aβ level fluctuation upon catheterization primarily depends on the sampling frequency and volume, but not on the catheterization procedure or inflammatory reaction. An optimized low-frequency sampling protocol minimizes or eliminates fluctuation of CSF Aβ levels, which will improve the capability of accurately measuring the pharmacodynamic read-out for amyloid-lowering therapies.

**Trial registration:**

ClinicalTrials.gov NCT01436188. Registered 15 September 2011.

**Electronic supplementary material:**

The online version of this article (doi:10.1186/s13195-016-0184-z) contains supplementary material, which is available to authorized users.

## Background

Alzheimer’s disease (AD) neuropathology is characterized by deposition in the brain of amyloid plaques, consisting mainly of amyloid-beta (Aβ) peptides, and neurofibrillary tangles, composed of hyperphosphorylated tau protein. Levels of cerebrospinal fluid (CSF) biomarkers Aβ and tau closely reflect the central pathogenic processes in AD and have proven their utility in evaluating disease risk or prognosis, guiding clinical diagnosis and monitoring therapeutic interventions [1, 2].

Aβ peptides are proteolytic cleavage products of amyloid precursor protein, formed by β- and γ-secretase activity. Aβ species predominantly include peptides of 1–40 amino acids (Aβ_1–40_) and 1–42 amino acids (Aβ_1–42_). Several studies report pronounced increases in CSF Aβ levels relative to baseline upon repeated CSF sampling with spinal catheters [3–9]. Additionally, methodological challenges in measuring Aβ in CSF are well described, but influencing factors are only partly understood [10–13].

Considering these challenges, there is a clear need to optimize and standardize the technique of continuous CSF sampling and measurement of CSF Aβ for application in clinical trials for Aβ-lowering compounds. We performed a study utilizing continuous CSF sampling with indwelling catheters over 36 h in healthy older participants to evaluate the effects on CSF Aβ levels when applying various sampling protocols. We investigated the impact of sampling frequency, volume, catheterization procedure, and pretreatment with an anti-inflammatory agent (ibuprofen) on changes in CSF Aβ levels over time. We also monitored the impact of different sampling schemes and CSF volume on the safety and tolerability of procedures.

## Methods

### Study population

Healthy men or women (*n* = 24; age 55–85 years, body mass index (BMI) 18–35 kg/m^2^) with Mini-Mental State Examination (MMSE) scores ≥27 were enrolled. None of these participants had any significant history of or clinically significant current medical illness at screening or admission, and neither did they have any sign of intracranial pressure as confirmed by fundoscopy.

Participants were excluded if they received the following drugs: aspirin (even low dose) within 5 days, low molecular weight heparin within 12 h, or anticoagulant treatment (besides the heparin already described) within 1 week before spinal catheterization (Additional file 1: Inclusion and exclusion criteria).

The study was conducted in accordance with the ethical principles that have their origin in the Declaration of Helsinki and that are consistent with current International Conference on Harmonization (ICH) guidelines on good clinical practices (GCP) and applicable regulatory requirements, and in compliance with the study protocol. The study protocol was reviewed and approved by the Institutional Review Board (Commissie voor Medische Ethiek, ZNA, Antwerp, Belgium). Written informed consent was obtained for participation and a separate consent for pharmacogenomic (DNA) sampling.

### Study design

**Fig. 1:**
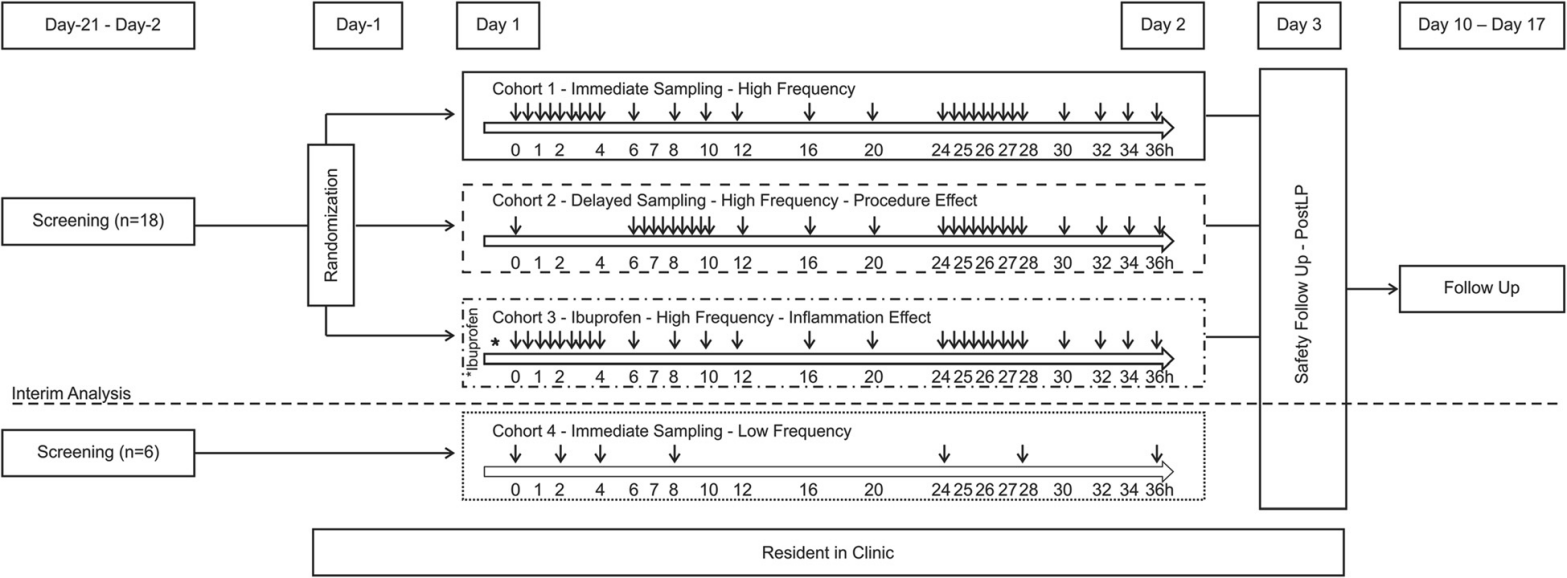
Study design. *Arrows* indicate time points of CSF sampling post spinal catheter placement. Cohort 1 and Cohort 3: 0, 0.5, 1, 1.5, 2, 2.5, 3, 3.5, 4, 6, 8, 10, 12, 16, 20, 24, 24.5, 25, 25.5, 26, 26.5, 27, 27.5, 28, 30, 32, 34, and 36 h. Cohort 2: 0, 6, 6.5, 7, 7.5, 8, 8.5, 9, 9.5, 10, 12, 16, 20, 24, 24.5, 25, 25.5, 26, 26.5, 27, 27.5, 28, 30, 32, 34, and 36 h. Cohort 4: 0, 2, 4, 8, 24, 28, and 36 h. Per time point, 6 ml of CSF was collected for cohorts 1–3 and 4 ml was collected for cohort 4. *LP* lumbar puncture

This open-label, single-center (Belgium), biomarker study (ClinicalTrials.gov NCT01436188) without investigational medicinal product, conducted between September 2011 and June 2013, consisted of a screening period (between 21 and 2 days before catheter insertion), an in-patient CSF collection period (1–3 days), and a follow-up period (~7–14 days after removal of the catheter). For each participant, the maximal study duration did not exceed 6 weeks (Fig. 1).

Initially participants (*n* = 18) were randomized into one of three cohorts (*n* = 6/cohort): cohort 1, high-frequency CSF sampling scheme with immediate sampling post catheterization; cohort 2, high-frequency CSF sampling scheme with delayed sampling, starting 6 h post catheterization; and cohort 3, high-frequency CSF sampling scheme with immediate sampling and prelumbar puncture treatment with ibuprofen on day 1 (single dose, 800 mg 2 h before catheterization). CSF was sampled at the following time points post spinal catheter placement for cohorts 1 and 3: 0, 0.5, 1, 1.5, 2, 2.5, 3, 3.5, 4, 6, 8, 10, 12, 16, 20, 24, 24.5, 25, 25.5, 26, 26.5, 27, 27.5, 28, 30, 32, 34, and 36 h. For cohort 2, the sampling time points were as follows: 0, 6, 6.5, 7, 7.5, 8, 8.5, 9, 9.5, 10, 12, 16, 20, 24, 24.5, 25, 25.5, 26, 26.5, 27, 27.5, 28, 30, 32, 34, and 36 h.

Following an interim review of the data from cohorts 1–3, six healthy older participants were allocated to an additional cohort: cohort 4, low-frequency CSF sampling scheme with immediate sampling. CSF was sampled at the following time points post spinal catheter placement for cohort 4: 0, 2, 4, 8, 24, 28, and 36 h (Fig. 1).

### CSF sampling

CSF samples (6 ml for cohorts 1–3 and 4 ml for cohort 4, per time point) were collected over 36 h using a lumbar spinal indwelling CSF catheter. On day 1, all participants received a lumbar indwelling CSF catheter (interspace of L3 and L4 or L4 and L5 of the lumbar spine) between 6:00 and 9:00 a.m. The introducer needle (Tuohy needle) with stylet in place was inserted at the superior aspect of the inferior spinous process in the midline and approximately 15° cephalad. The stylet was removed and the peridural space was entered using a loss of resistance technique. A Spinocath Catheter (22 Gauge; BBraun Melsungen AG, Germany) with a spinal needle inside was introduced through the Tuohy needle to perforate the dura mater (“dural pop”). The spinal needle was withdrawn while moving up the spinal catheter into the subarachnoid space for 10–15 cm (distance tip to skin). The Tuohy needle was subsequently removed. To reduce the risk of CSF leakage, participants were preferably placed on a bed, resting in a supine position during sampling and for up to 12–24 h after removal of the catheter. No specific cognitive activity was mandated, but most participants were reading or listening to music.

CSF samples were collected with a syringe (BD Plastipack 2 and 5 ml) under sterile conditions from the moment of catheter placement (0 h or baseline sample) through the 36-h assessment period. Samples were collected in 10 ml (catalog number 62.610.018)/12 ml (catalogue number 60.541.004) Sarstedt Liquor tubes, which were immediately placed on melting ice and gently inverted for adequate mixing. The collected CSF volume was aliquoted by immediate transfer of 500 μl samples to multiple storage tubes (Micronic 1.4 ml noncoded U-bottom tubes in Comorack-96 (catalogue number MP22502) with caps from FluidX (Split TPE Capcluster Blue, catalogue number 65-53028)) and stored at −70 °C.

All sampling and processing materials used were analyzed for their Aβ adsorption capacity and were found acceptable (<20 %) for avoiding any major influence on the Aβ read-outs (unpublished data).

### Analysis of CSF Aβ levels

A qualified, specific, and sensitive prototype multiplex immunoassay developed by Janssen Research & Development and based on Meso Scale Discovery (MSD) electrochemiluminescence detection technology was utilized for simultaneous detection of four CSF Aβ species (Aβ_1–37_, Aβ_1–38_, Aβ_1–40_, and Aβ_1–42_) [14, 15]. Purified monoclonal antibodies specific for Aβx-37 (JRD/Aβ37/3), Aβx-38 (J&JPRD/Aβ38/5), Aβx-40 (JRF/cAβ40/28), and Aβx-42 (JRF/cAβ42/26) were coated on MSD 4-plex 96-well plates as capture antibodies. JRF/AβN/25, which displays specificity for Aβ isoforms with intact N-terminus (i.e., full-length Aβ), was utilized as the detection antibody [16]. The CSF Aβ_1–37_, Aβ_1–38_, Aβ_1–40_, and Aβ_1–42_ concentrations were determined using a standard curve with a four-parameter logistic model with 1/*Y*^2^ weighting function.

One operator performed all of the experimental analyses for participants in cohorts 1–3, in a random fashion over 3 consecutive days. The experimental analysis for cohort 4 was performed by one operator on the same day. All samples from each participant were analyzed in duplicate on the same plate. Only mean values with a replicate well coefficient of variation (CV) ≤20 % were accepted.

### Analysis of baseline CSF Aβ_1–42_, T-tau, and phosphorylated tau_181P_ levels

Baseline Aβ_1–42_, P-tau_181P_, and total tau (T-tau) concentrations (i.e., directly after catheter insertion) were measured utilizing INNO-BIA AlzBio3 kit reagents and the Luminex analytical platform [17]. Diagnostic threshold CSF concentrations for AD versus normal controls for Aβ_1–42_ were applied to the current sample set to judge the likelihood of having cerebral amyloid plaque deposition [18].

### Apolipoprotein E (*APOE*) epsilon 4 carrier status

Blood samples (10 ml) were collected in tubes containing potassium/sodium EDTA. DNA was isolated using Puregene chemistry and automated extraction using an Autopure LS. The aliquots of DNA samples from all participants were genotyped in a multiplex reaction using PCR/ligation detection reaction [19].

### Safety assessments

Adverse events (AEs) were monitored. Laboratory tests, examination of vital signs, resting 12-lead ECGs, physical and neurological examinations, and fundoscopy were performed.

### Sample size

Based on CSF determinations of Aβ_1–42_, Aβ_1–40_, and Aβ_1–38_ with a similar enzyme-linked immunosorbent assay (ELISA) method (Janssen, unpublished data), the CV of percentage change from baseline was estimated to be ~13 %. A sample size of five completers per group was expected to produce two-sided 95 % confidence intervals with 16 % precision compared with natural fluctuation of around 15 % in the reference study (ClinicalTrials.gov NCT01556217) and was considered reasonable for precise estimation of mean.

## Results

### Demographics, baseline characteristics, and AD biomarker pattern

Twenty-four participants were enrolled, most were men with mean age of 64 years. The average measures for age, weight, height, BMI, and MMSE score at screening were comparable among cohorts (*p* = 0.42, 0.16, 0.56, 0.66, and 0.45, respectively, *F* test) (Table 1). AD biomarkers were analyzed. Two participants had Aβ_1–42_ below the threshold concentration (≤249 pg/ml) [18], suggested to be pathologic, but none had elevated T-tau or P-tau_181P_ values (Additional file 2: Figure S1). Hence, no participant had a typical AD biomarker pattern. All participants completed the study.

**Table 1:** Demographics and baseline characteristics of study participants by cohort (all randomized participants)

	Cohort 1	Cohort 2	Cohort 3	Cohort 4	Total
	(*n* = 6)	(*n* = 6)	(*n* = 6)	(*n* = 6)	(*N* = 24)
Age (years)	62 (4.3)	64 (6.4)	66 (1.7)	63 (3.3)	64 (4.3)
Sex (men), *n* (%)	6 (100.0)	5 (83.3)	6 (100.0)	5 (83.3)	22 (91.7)
Race, *n* (%)					
White	6 (100.0)	6 (100.0)	5 (83.3)	6 (100.0)	23 (95.8)
Black or African American	0	0	1 (16.7)	0	1 (4.2)
Ethnicity, *n* (%)					
Not Hispanic or Latino	6 (100.0)	6 (100.0)	6 (100.0)	6 (100.0)	24 (100.0)
Weight (kg)	83 (10.8)	72 (8.3)	77 (5.6)	79 (10.6)	78 (9.6)
Height (cm)	176 (7.8)	170 (9.5)	171 (9.2)	173 (7.6)	172 (8.4)
BMI (kg/m^2^)	27.1 (3.8)	25 (2.3)	26.6 (2.7)	26.6 (3.5)	26.3 (3.0)
Total MMSE at screening	29.2 (0.8)	29.0 (1.3)	29.7 (0.5)	29.7 (0.8)	29.4 (0.9)
Aβ_1-42_ (pg/ml)	393.2 (26.4)	380.6 (44.1)	326.1 (75.4)	319.2 (97.6)	354.8 (70.6)
T-tau (pg/ml)	74.5 (20.0)	62.7 (28.9)	46.7 (20.6)	50.7 (21.9)	58.6 (24.3)
P-tau_181P_ (pg/ml)	30.7 (8.2)	22.2 (4.9)	20.4 (6.9)	24.9 (13.0)	24.6 (9.1)
*APOE* ε4 carrier					
Yes	1 (16.7)	1 (16.7)	0	2 (33.3)	4 (16.7)
No	4 (66.7)	3 (50.0)	5 (83.3)	4 (66.7)	16 (66.7)
Unknown	1 (16.7)	2 (33.3)	1 (16.7)	0	4 (16.7)

Data shown as mean (standard deviation), unless otherwise specified

Cohort 1: immediate sampling, high frequency; cohort 2: delayed sampling, high frequency, procedure effect; cohort 3: ibuprofen, high frequency, inflammation effect; cohort 4: immediate sampling, low frequency. *APOE*, apolipoprotein E gene; *BMI*, body mass index; *MMSE*, mini–mental state examination; *Aβ*, amyloid beta; *P-tau*, phosphorylated tau; *T-tau*, total tau

### CSF Aβ levels

#### Cohort 1: high-frequency CSF sampling scheme with immediate sampling

**Fig. 2:**
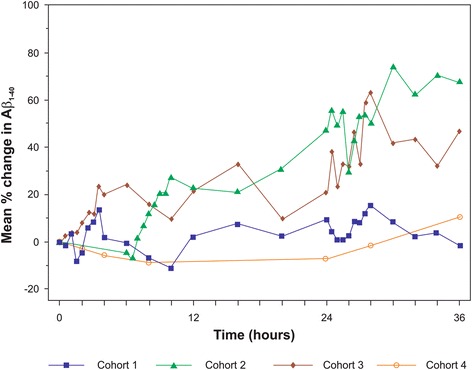
Mean % change from baseline of CSF Aβ_1–40_ for all cohorts. Standard deviation is not shown for clarity of representation. Individual profiles can be found in Fig. 3. Cohort 1: immediate sampling, high frequency; cohort 2: delayed sampling, high frequency, procedure effect; cohort 3: ibuprofen, high frequency, inflammation effect; cohort 4: immediate sampling, low frequency. *Aβ* amyloid beta

**Fig. 3:**
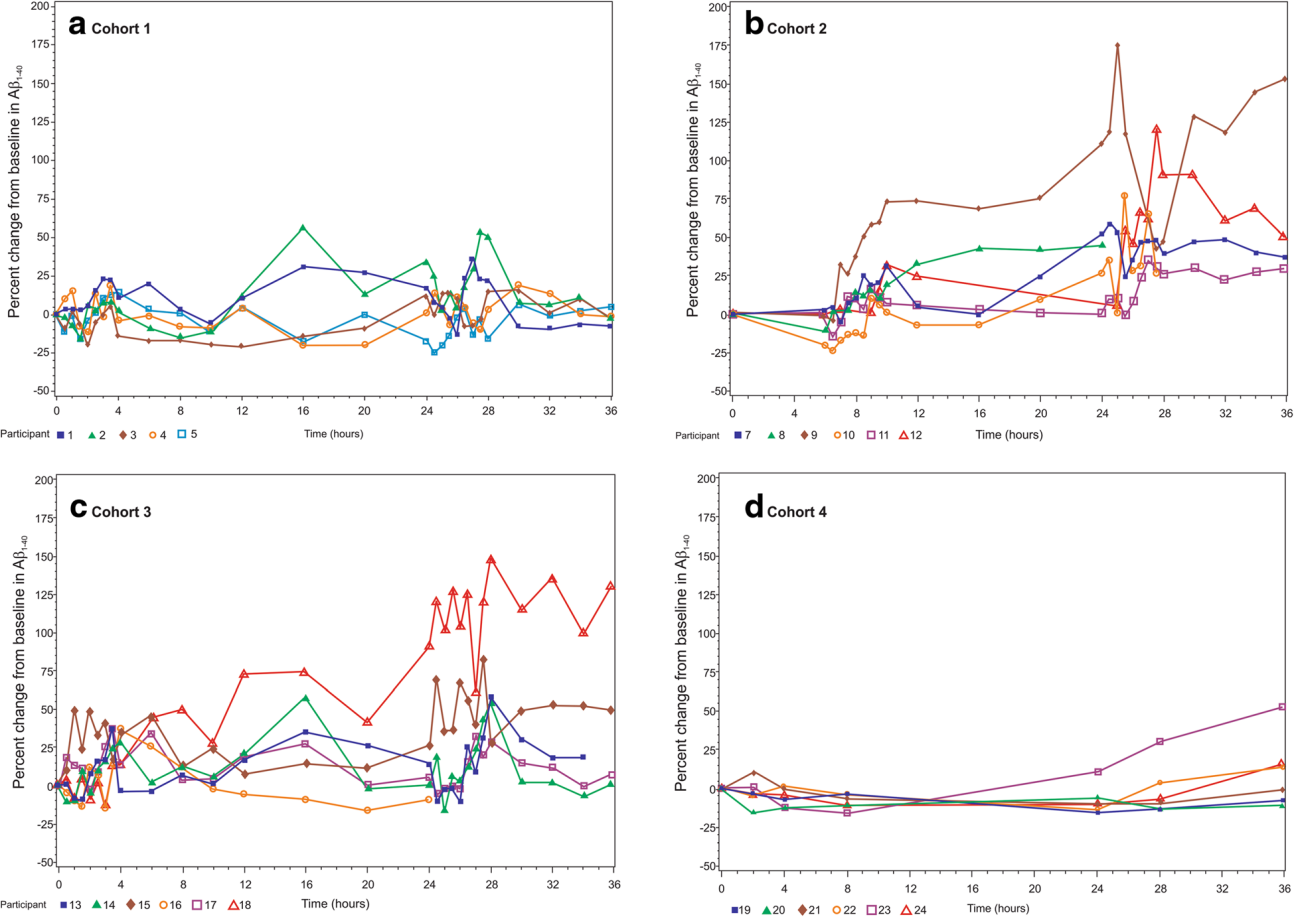
Individual CSF Aβ_1–40_ profiles for all participants per cohort. **a** Cohort 1: immediate sampling, high frequency. **b** Cohort 2: delayed sampling, high frequency, procedure effect. **c** Cohort 3: ibuprofen, high frequency, inflammation effect. **d** Cohort 4: immediate sampling, low frequency. *APOE* ε4 carriers are indicated in *red* with a *square* or *diamond* symbol. Cohort 1: only five participants are shown; one participant (participant 6) did not have a baseline sample available. *Aβ* amyloid beta (Color figure online)

Mean CSF Aβ_1–40_ levels fluctuated with a tendency for increasing levels relative to baseline over the sampling period (Fig. 2). CSF Aβ levels were particularly increased during periods of frequent sampling (sampling every 30 min: 0–4 h and 24–28 h). After the 12-h time point, two participants showed increases in CSF Aβ_1–40_ levels of >20 % at several time points compared with baseline. Other participants had a rather stable pattern of CSF Aβ over the 36 h (Fig. 3a).

#### Cohort 2: high-frequency CSF sampling scheme with delayed sampling

In contrast to cohort 1, mean CSF Aβ_1–40_ levels at baseline and 6 h post catheterization were similar in cohort 2 (Fig. 2). CSF Aβ levels increased in periods of frequent sampling (sampling every 30 min: 6–10 h and 24–28 h). Notably in cohort 2, unlike cohort 1, CSF Aβ levels remained elevated after the first period of frequent sampling. The elevation of mean Aβ levels was not limited to the second period of frequent sampling (24–28 h), but started before this period and extended beyond it. Fluctuation of mean CSF Aβ_1–40_ over 36 h was higher in cohort 2 than in cohort 1. Increases in CSF Aβ over 36 h were observed for all participants in cohort 2. After the 10-h time point, one participant showed increased CSF Aβ_1–40_ levels of >60 % at many subsequent time points (Fig. 3b).

#### Cohort 3: high-frequency CSF sampling scheme with immediate sampling and ibuprofen pretreatment

Mean CSF Aβ_1–40_ levels in this cohort showed more fluctuation over 36 h than that observed for cohort 1 (Fig. 2). Similarly to cohorts 1 and 2, CSF Aβ levels increased during periods of frequent sampling. Similar to cohort 2, the CSF Aβ levels remained elevated after the first period of frequent sampling. During the first period of frequent sampling (0–4 h), all participants, except one who showed the earliest increases in CSF Aβ levels, had increased levels between 3 and 4 h. One participant showed an increase in CSF Aβ_1–40_ levels >60 % (after 12 h) and >100 % (after 25 h) (Fig. 3c).

Several studies support γ-secretase modulator activity of ibuprofen [20, 21]. Mean baseline CSF Aβ_1–42_ levels were not significantly reduced in the ibuprofen-treated cohort versus the other cohorts (Additional file 2: Figure S1; *p* = 0.26, *F* test), suggesting that 800 mg ibuprofen did not lower baseline CSF Aβ_1–42_ levels.

#### Cohort 4: low-frequency CSF sampling scheme with immediate sampling

Mean CSF Aβ_1–40_ levels over 36 h were more stable in this cohort versus the high-frequency sampling schemes (Fig. 2). Only one participant showed a pronounced increase in the CSF Aβ_1–40_ levels, observed after the 28-h time point (Fig. 3d).

For all cohorts, Aβ_1–37_, Aβ_1–38_, and Aβ_1–42_ were measured in addition to Aβ_1–40_ and similar profiles were noted for mean change in Aβ levels versus baseline (data not shown). The Aβ ratios showed lower intrasubject variability than that observed for individual peptides over time, suggesting that the effect of different sampling schemes on CSF Aβ levels was similar for Aβ_1–37_, Aβ_1–38_, Aβ_1–40_, and Aβ_1–42_.

### Possible sources of intersubject variability

Intersubject variability was noted in all cohorts relating to increase in CSF Aβ levels over time. No correlation was observed between increases in CSF Aβ levels and sex, age, *APOE* ε4 status, MMSE score, CSF sampling or sample processing issues, or baseline levels of Aβ_1–42_, P-tau_181P_, and T-tau (Additional file 3: Table S1).

A trend for a higher incidence of headache (10/15 (66.7 %)) was observed in participants with an Aβ increase of >25 % versus baseline compared with participants with an Aβ increase <25 % (2/8 (25 %)) (Fisher’s exact test: *p* = 0.089).

### Safety and tolerability of CSF sampling procedures

Nineteen (79.2 %) participants experienced at least one AE, which was mild or moderate in severity, and the majority of events were resolved at follow-up. Incidence of AEs was not significantly different across cohorts (*p* = 0.11, Fisher’s exact test). Headache (*n* = 12 (50.0 %)), back pain (*n* = 7 (29.2 %)), and catheter-site pain (*n* = 3 (12.5 %)) were most frequently reported, and were possibly related to the sampling procedure (Table 2). There was only one occurrence (4.2 %) of postdural puncture headache. No clinically relevant changes in vital signs, ECG, or laboratory measurements were noted. There were no signs of inflammation as indicated by clinical laboratory measurements.

**Table 2:** Incidence of adverse events occurring during study (starting after catheter insertion)

	Cohort 1	Cohort 2	Cohort 3	Cohort 4	Total
	(*n* = 6)	(*n* = 6)	(*n* = 6)	(*n* = 6)	(*N* = 24)
Participants with one or more adverse event	6 (100.0)	6 (100.0)	3 (50.0)	4 (66.7)	19 (79.2)
Headache	3 (50.0)	5 (83.3)	2 (33.3)	2 (33.3)	12 (50.0)
Back pain	1 (16.7)	2 (33.3)	1 (16.7)	3 (50.0)	7 (29.2)
Catheter site pain	2 (33.3)	0	1 (16.7)	0	3 (12.5)
Dizziness postural	0	1 (16.7)	0	1 (16.7)	2 (8.3)
Hypoesthesia	1 (16.7)	0	1 (16.7)	0	2 (8.3)
Pain in extremity	1 (16.7)	0	1 (16.7)	0	2 (8.3)
Nausea	0	1 (16.7)	1 (16.7)	0	2 (8.3)
Musculoskeletal stiffness	0	1 (16.7)	0	0	1 (4.2)
Neck pain	0	0	1 (16.7)	0	1 (4.2)
Gastroesophageal reflux disease	0	0	1 (16.7)	0	1 (4.2)
Regurgitation	0	0	0	1 (16.7)	1 (4.2)
Toothache	0	0	0	1 (16.7)	1 (4.2)
Dermatophytosis	0	0	1 (16.7)	0	1 (4.2)
Sinusitis	0	0	0	1 (16.7)	1 (4.2)
Post lumbar puncture syndrome	0	1 (16.7)	0	0	1 (4.2)
Pruritus	1 (16.7)	0	0	0	1 (4.2)
Flushing	0	1 (16.7)	0	0	1 (4.2)

Data shown as *n* (%). Cohort 1: immediate sampling, high frequency; cohort 2: delayed sampling, high frequency, procedure effect; cohort 3: ibuprofen, high frequency, inflammation effect; cohort 4: immediate sampling, low frequency

The laboratory parameters (clinical chemistry, hematology, urinalysis) were evaluated at screening, on days –1 and 2, and during follow-up (days 7–14 after removal of the spinal catheter). The vital signs and resting 12-lead ECGs were evaluated at screening, on days –1, 1, and 2, and during follow-up. The participants were physically and neurologically examined at screening, on days 1 and 3, and in follow-up. Fundoscopy was performed at screening to exclude intracranial pressure

## Discussion

CSF Aβ concentrations were shown to be stable over months to years in longitudinally collected samples with isolated lumbar punctures [22, 23]. However, the intrasubject CSF Aβ_1–40_ and Aβ_1–42_ levels varied largely and tended to rise over 36 h after hourly sampling via an intrathecal catheter in healthy participants [5]. Consistent with other studies, an increase in CSF Aβ levels following serial samplings was noted in our study [3, 4, 6, 7, 9]. Factors such as frequency of sampling and sample volume [4], diurnal effects [5, 7, 8], catheter interaction with CSF Aβ at the first sampling time points, and activity [5, 7] were suggested to be related to this observed increase. However, further studies to delineate the exact mechanisms are needed.

We evaluated the effect of different CSF sampling protocols on CSF Aβ levels in an older population to reflect the age range of the target population for AD. Earlier studies mainly describe CSF Aβ changes for younger healthy participants. A study comparing data from older healthy controls and AD patients showed that the variation in CSF measures of Aβ_1–40_, Aβ_1–42_, T-tau, and P-tau_181P_ was comparable [8]. On the other hand, hour-to-hour fluctuations in CSF biomarkers were lower in older healthy controls and AD patients than in young participants [5], underscoring the importance of investigating these changes in the target population.

Our results corroborated earlier findings indicating a higher increase in CSF Aβ levels with protocols using high sampling frequency compared with protocols using low sampling frequency [4]. The increases in Aβ levels relative to baseline were more prominent during high-frequency sampling (every 30 min) in cohorts 1–3, while the Aβ levels were relatively stable in samples drawn every 4 h. Although the first period of frequent sampling occurs at a different time during the day for cohort 2 compared with cohorts 1 and 3, clear mean increases in CSF Aβ levels were seen in high-frequency sampling periods for all cohorts. Together with the fact that the average Aβ levels did not return to baseline after 24 h, this suggests that the observed increase in our study could not be explained solely by a diurnal effect.

Interestingly, Aβ levels at baseline and 6 h post catheterization were similar in cohort 2, suggesting that the increase in Aβ levels was not related to the catheter insertion procedure. Furthermore, precatheterization administration of ibuprofen did not impact the increased CSF Aβ levels. These findings indicate that the increase in CSF Aβ levels was not related to the procedure of catheterization itself, including any kind of induced inflammatory reaction. Together, these data support the hypothesis that the intense sampling frequency applied in these cohorts probably impacted CSF Aβ levels.

After the interim review of the data from cohorts 1–3, it was hypothesized that a lower CSF sampling frequency over 36 h may result in more stable Aβ profiles. An additional cohort (cohort 4) of healthy older participants was included to assess an alternative low-frequency CSF sampling scheme. This sampling scheme, with a lower total volume of CSF sampled, resulted in more stable mean CSF Aβ levels over 36 h versus high-frequency sampling schemes. These data support the hypothesis that the increase in CSF Aβ levels compared with baseline level is probably related to either CSF sampling frequency or volume, or both. The underlying mechanism of the rise in CSF Aβ concentrations after frequent CSF sampling is unknown. Various stimuli, including stress and sleep deprivation, could increase brain Aβ levels, possibly through modulation of neuronal activity [24]. Moreover, frequent sampling in the lumbar region might alter a possible rostral–caudal Aβ gradient from ventricular to lumbar CSF by induction of redistribution of CSF to the lumbar region [4]. The disease state may also influence the CSF Aβ levels and thus studies in AD patients are warranted [8].

One out of six participants in cohort 4 demonstrated a rise in CSF Aβ levels after 24 h post catheterization. This increase does not seem to be related to gender, age, race, APOE status, MMSE score, or adverse events (Additional file 3: Table S1). As such, a possible explanation for this finding is currently unclear, but might for example include a difference in activity level of this participant. Only one participant in cohort 4 had an Aβ_1–42_ concentration slightly below the threshold concentration, excluding the possibility that greater amyloid deposition in cohort 4 would be responsible for the observed stability of CSF Aβ concentrations over time in this cohort [25].

## Conclusions

CSF Aβ levels were substantially affected by CSF sampling frequency and/or sampling volume. An optimized sampling protocol with lower CSF sampling frequency and volume resulted in more stable Aβ profiles. In future clinical studies with Aβ-targeting experimental drugs, this protocol would lead to a better estimation of drug effects on Aβ levels after continuous CSF sampling and thus lower the required sample size. The described protocols for continuous CSF sampling were well tolerated with no clinically important safety concerns in healthy older participants. These results substantiate the potential of CSF Aβ as a pharmacodynamic biomarker using frequent CSF sampling to assess evidence of target engagement and pharmacological activity of Aβ-targeting compounds.

### Statistical analysis

JB was the project statistician (Janssen Research & Development, LLC, Raritan, NJ, USA).

### Ethics approval

Commissie voor Medische Ethiek, ZNA, Antwerp, Belgium (Institutional Review Board) approved the study.

## Additional files

Additional file 1: Details the inclusion and exclusion criteria of the study. (PDF 7 kb) Additional file 2: Figure S1 showing scatter plots representing measures of individual participants for baseline concentrations of CSF Aβ_1–42_, P-tau_181P_, and T-tau per cohort. (PDF 131 kb) Additional file 3: Table S1 presenting an overview of individual participant characteristics at baseline. (PDF 97 kb)

## Abbreviations

AD: Alzheimer’s disease
AE: adverse event
APOE: apolipoprotein E
Aβ: amyloid beta
BMI: body mass index
CSF: cerebrospinal fluid
CV: coefficient of variation
ELISA: enzyme-linked immunosorbent assay
GCP: Good Clinical Practices
ICH: International Conference on Harmonization
MMSE: Mini-Mental State Examination
MSD: Meso Scale Discovery
P-tau: phosphorylated tau
T-tau: total tau
