# Supplementary material for: Impact of frequent cerebrospinal fluid sampling on Aβ levels: systematic approach to elucidate influencing factors
Source: Alzheimers Res Ther. 2016 May 19;8:21. doi: 10.1186/s13195-016-0184-z (PMC4875639; doi:10.1186/s13195-016-0184-z)
Supplement: Additional file 1: — Details the inclusion and exclusion criteria of the study. (PDF 7 kb) [file 13195_2016_184_MOESM1_ESM.pdf]

Additional file 1: Inclusion and exclusion criteria

Inclusion:

Postmenopausal (for at least 12 months) women. Healthy participants assessed by physical examination, clinical laboratory tests, medical history, vital signs, and ECG. Participants willing to refrain from smoking or the use of nicotine-based products from day 1 until at least 12h after removal of the spinal catheter.

Exclusion:

Participants having relevant history of lower back pain or scoliosis and/or major (lumbar) back surgery; history of severe bleeding with unclear origin; history of epilepsy; history or family history of abnormal bleeding/blood clotting; history or family history of anemia; history of or current neurological disease other than AD/MCI (including any history of postdural puncture headache) were excluded.

Participants were excluded for any clinically significant abnormality in physical/neurological examination, vital signs, or ECG; or if clinically significant acute illness within 7 days before study drug administration.

Participants were also excluded if they were: allergic to local anesthetics and/or chlorhexidine; hypersensitive, or intolerant to ibuprofen or one of its excipients and/or has any contraindication for ibuprofen; positive for hepatitis B surface antigen, hepatitis C antibodies or HIV antibodies.
