# Supplementary material for: Impact of frequent cerebrospinal fluid sampling on Aβ levels: systematic approach to elucidate influencing factors
Source: Alzheimers Res Ther. 2016 May 19;8:21. doi: 10.1186/s13195-016-0184-z (PMC4875639; doi:10.1186/s13195-016-0184-z)
Supplement: Additional file 2: — Figure S1 showing scatter plots representing measures of individual participants for baseline concentrations of CSF Aβ1–42, P-tau181P, and T-tau per cohort. (PDF 131 kb) [file 13195_2016_184_MOESM2_ESM.pdf]

Additional file 2: Figure S1: Scatter plots of baseline concentrations of CSF Aβ_{1-42}, P-tau$_{181P}$ and T-tau per cohort.

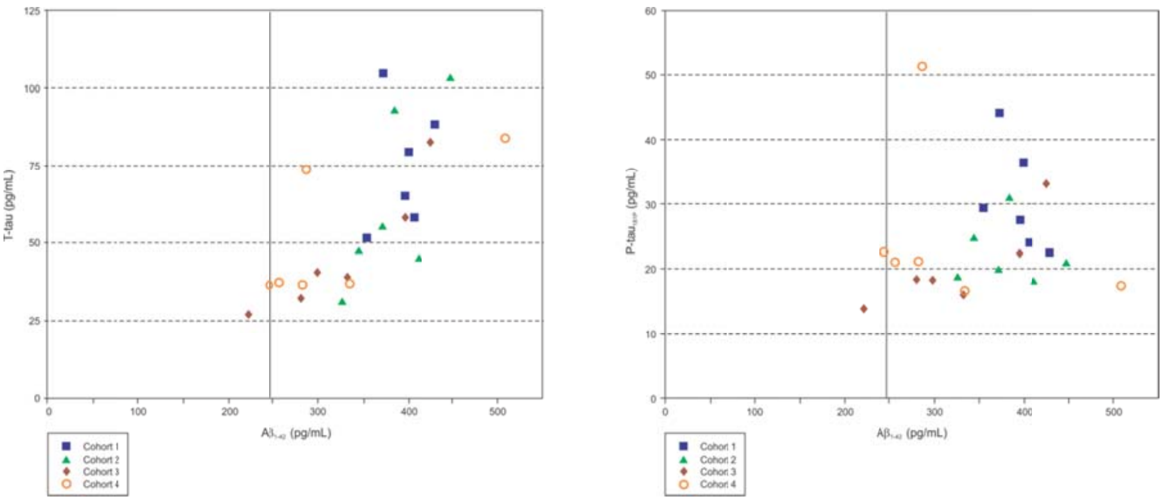

Analyses were performed utilizing INNO-BIA AlzBio3 kit reagents and the Luminex analytical platform. Measures for individual participants are indicated with symbols referring to the corresponding cohort. Diagnostic threshold CSF concentration for Alzheimer's disease versus normal controls for Aß$_{1-42}$ (≤249 pg/mL) is indicated as a vertical line on the plot.

Cohort 1: immediate sampling – high frequency; cohort 2: delayed sampling – high frequency – procedure effect; cohort 3: ibuprofen – high frequency – inflammation effect; cohort 4: immediate sampling – low frequency
