# Supplementary material for: Impact of frequent cerebrospinal fluid sampling on Aβ levels: systematic approach to elucidate influencing factors
Source: Alzheimers Res Ther. 2016 May 19;8:21. doi: 10.1186/s13195-016-0184-z (PMC4875639; doi:10.1186/s13195-016-0184-z)
Supplement: Additional file 3: — Table S1 presenting an overview of individual participant characteristics at baseline. (PDF 97 kb) [file 13195_2016_184_MOESM3_ESM.pdf]

Additional file 3: Table S1: Overview of individual participant characteristics at baseline

Cohort	Participant	Maximum CSF $A\beta_{1-40}$ %Change From Baseline	*APOE* Status	[a]Total MMSE Score	CSF Comments	$A\beta_{1-42}$ (pg/mL)	P-tau_{181P} (pg/mL)	T-tau (pg/mL)	Adverse events
Cohort 1	1	36.05	ε 3/ ε 3	30		399.9	36.5	79.4	Headache
	2	56.23	ε 3/ ε 3	29		395.6	27.6	65.2	Headache
	3	15.79	ε 3/ ε 3	29		372.8	44.2	104.6	Back pain, pruritus, headache
	4	19.69	ε 3/ ε 4	29		354.8	29.4	51.5	Catheter site pain, hypoesthesia
	5	14.18	ε 3/ ε 3	30		430.2	22.5	88.2	Pain in extremity
	6			28	No sample could be taken at baseline (0h)	406.1	24.0	58.2	Catheter site pain
Cohort 2	7	58.71	ε 2/ ε 3	27		447.6	20.8	103.4	Headache
	8	44.78		30	No CSF samples available after 24h	410.3	18.2	45.3	Back pain, headache
	9	174.58	ε 3/ ε 3	30	No CSF sample at 26h, 26h 30min, 27h (volume low)	326.3	18.7	31.2	Musculoskeletal stiffness, headache, back pain
	10	77.40		29	No CSF samples available after 27.5 h	383.8	30.9	93.2	Headache, dizziness postural
	11	34.73	ε 3/ ε 3	28		344.7	24.6	47.4	Headache, nausea, post lumbar puncture syndrome

	12	119.76	ε 3/ ε 4	30	No CSF samples available (7h 30min, 8h, 8h 30min, 20h, 24h, 24h 30min)<br>No CSF sample at 16h	370.7	19.8	55.7	Flushing
Cohort 3	13	57.49	ε 3/ ε 3	30		281.4	18.4	32.4	
	14	57.83		30		425.6	33.3	82.7	
	15	82.22	ε 2/ ε 3	30		332.7	15.9	39.2	Headache, dermatophytosis, gastroesophageal reflux disease, pain in extremity
	16	36.59	ε 3/ ε 3	30	No CSF samples available after 24 h	299.3	18.4	40.4	Catheter site pain, hypoaesthesia
	17	37.00	ε 3/ ε 3	29		395.7	22.4	58.3	
	18	148.12	ε 3/ ε 3	29		221.7	14.1	27.0	Back pain, headache, neck pain, nausea
Cohort 4	19	-3.97	ε 3/ ε 3	30		508.5	17.3	83.8	Back pain, headache
	20	-6.32	ε 3/ ε 4	28		287.2	51.1	73.4	
	21	10.51	ε 3/ ε 3	30		334.1	16.6	36.7	Back pain
	22	13.72	ε 3/ ε 4	30		257.2	21.0	37.1	
	23	52.86	ε 3/ ε 3	30		282.7	21.1	36.5	Headache
	24	16.32	ε 3/ ε 3	30		245.7	22.5	36.5	Dizziness postural, regurgitation, back pain, sinusitis, toothache

[a] at screening. Cohort 1: immediate sampling – high frequency; cohort 2: delayed sampling – high frequency – procedure effect; cohort 3: ibuprofen – high frequency – inflammation effect; cohort 4: immediate sampling – low frequency. M, men; W, women; *APOE,* apolipoprotein E gene; BMI, body mass index; CSF, cerebrospinal fluid; MMSE, mini-mental state examination
